# Editorial: Innovations and Perspectives in Data Mining and Knowledge Discovery

**DOI:** 10.3389/fdata.2020.637906

**Published:** 2021-01-18

**Authors:** Naoki Abe, Huan Liu, Kuansan Wang

**Affiliations:** ^1^ IBM Research, Yorktown Heights, NY, United States; ^2^ Computer Science & Engineering, Arizona State University, Tempe, AZ, United States; ^3^ Microsoft Research, Redmond, WA, United States

**Keywords:** Datamining, Bigdata, data analytics, big data, knowledge discovery

In this edited collection under a special research topic, we present four recent articles in the broader areas surrounding data mining and knowledge discovery for big data, which together attest to the diversity and broadness of application domains and associated technical agenda being explored in this fast evolving discipline. In particular, the collection centers around papers affiliated with the ACM SIGKDD International Conference on Data Mining and Knowledge Discovery (KDD 2019) and its satellite workshops (e.g., WSDM: Workshop on Issues of Sentiment Discovery and Opinion Mining, Fragile Earth: Data Science for a Sustainable Planet), which address a varying set of agenda such as epistemology of data mining (e.g., societal and ethical aspects), automation of data mining (e.g., robustness of deep neural architecture search), advances in text mining (e.g. deep learning based embeddings of text data and data collection) and application in climate change mitigation (e.g., data collection eco-systems). It is our hope that this collection will provide the audience with a glimpse into the rich landscape of data mining related research agenda and applications of big data today.

The first article is on “Big Data and the Little Big Bang: An Epistemological (R)evolution” by Dominik Balazka and Dario Rodighiero. This is a timely article that looks at the field of big data in a refreshing perspective. The authors try to answer intriguing questions such as “What qualifies as big data? What does big data promise? Is big data a revolution or evolution?”. Starting from an analysis of frequently employed definitions of big data, the authors argue that there are intrinsic weaknesses of big data and it is more appropriate to define big data in relational terms. The excessive emphasis on volume and technological aspects of big data, combined with neglected epistemological issues, implies that big data is neutral, omni-comprehensive and theory free. The authors show that this rhetoric contradicts the empirical reality of big data: 1) data collection is not neutral nor objective; 2) big data has more data than before, but more does not mean all, the totality of the data population; and 3) interpretation and knowledge production remain both theoretically informed and subjective. The authors then argue that big data may be interpreted as a methodological revolution carried over by evolutionary processes in technology and epistemology, or a third paradigm. They also point out that big data has promoted a new digital divide between big data rich and big data poor populations, therefore, radically shaping the power dynamics involved in the processes of production and analysis of data.

The second article is about “On Robustness of Neural Architecture Search Under Label Noise” by Yi-Wei Chen, Qingquan Song, Xi Liu, P. S Sastry and Xia Hu. This article addresses the robustness against training data disturbances for neural networks. In recent years, we have seen explosive advancements in machine learning applications based on deep neural network (DNN) architecture. DNN is particularly appealing because, by jointly optimizing the feature extraction and the model parameter estimation, the framework can eliminate the need for manually crafting features and the mismatches between the feature design and the model training. The historical weakness of DNN in requiring massive supervision data is being alleviated by the advent of Big Data as ingenious ways are developed to adapt many easily accessible large datasets for light- or distant- supervision training. Amidst the flurry of successful DNN applications, however, lies a very fundamental question in the robustness of these systems against inevitable imperfection in their training data. The question is not only of theoretical interest but has become an urgent and grave concern in our society, where even more machine learning systems are deployed to assist decision making processes in broad areas ranging from credit assessment to public safety with consequential outcomes. This article tackles this question by examining the robustness issue through a Bayesian risk minimization point of view. The authors show that the core DNN training algorithms, based on backpropagating Euclidean errors, can often be augmented to conform to an objective function that can be mathematically proven to be more robust against noise corruptions in the examples. The theoretical results are backed by experiments on an image search task where the training data are deliberately injected with random noise at the pixel level. As similar computer vision technologies are now being used to sign in to our computers and by security authorities at the airports and sports stadiums, the insights unlocked by this research could not have waited any longer.

The third article is titled “Unsupervised Word Embedding Learning by Incorporating Local and Global Contexts” by Yu Meng, Jiawei Han (Corresponding authors), and their coauthors. Word embedding is a popular and effective technique that has benefited a broad spectrum of text analysis tasks. It learns distributed word representations to encode word semantics. Word embedding produces word representations by modeling local contexts of words, assuming that words are semantically close to surrounding words. In their article, the authors suggest that local contexts only partially define word semantics in the unsupervised word embedding learning; and global contexts, referring to the broader semantic units (e.g., the document or paragraph where the word appears), may be useful in capturing different aspects of word semantics and complement local contexts. They then propose two simple unsupervised word embedding models that jointly model both local and global contexts to learn word representations and show that they are effective. What is unique of this article is that the authors further provide theoretical interpretations of the proposed models to demonstrate how local and global contexts can be jointly modeled. Conducting a thorough evaluation on a wide range of benchmark datasets, they show that their two proposed models achieve superior performance on text classification tasks.

The fourth and final article is on “Next-Generation Digital Ecosystem for Climate Data Mining and Knowledge Discovery: A Review of Digital Data Collection Technologies” Angel Hsu, Willie Khoo, Nihit Goyal and Martin Wainstein. This article addresses the extremely timely and important question of how to enable data mining technologies in combating climate change, the “defining challenge” of our time. Despite the recognition that measuring and monitoring climate change and mitigation progress will be key in devising effective mitigation policies, there are still “data gaps” that hinder the scientists and policy makers from implementing them. In this review article, the authors examine the extent to which the emerging digital data collection technologies, such as Earth Observations (EO) and Internet-of-Things (IoT), can help address these gaps. They assess the promising potential of EO and IoT technologies, and point to current limitations and challenges, e.g. with regard to geographically disproportionate levels of data availability, inaccuracy and incompleteness of data, lack of methodological consistency in measurement and calculation, etc. They then discuss areas of on-going efforts to address them, such as standardization, data resolution, certainty and transparency. Finally, the article proceeds to discuss aspects of the next generation climate change tracking eco-system, including the use of Distributed Ledger Technology (DLT) to realize greater transparency and address data privacy concerns, aiming for comprehensive data governance including incentive mechanisms, policy agreement, etc.

Within the broad spectrum of research agenda of big data, this collection reflects the increasing diversity in the field of data mining and management in terms of both disciplines (communications, media and society, public policy, computer science and electrical engineering) and geography (Europe, Asia and North America). We expect this trend to continue or accelerate in the future, stimulated by the growing data science applications that have become commonplace in our daily lives. While it is a time of exhilaration, it is also a time of humility to all of us data scientists as our productions may exert ever more impact on our societies, with possible unintentional consequences. There seems no day that has gone by without incidents reminding us that data can be misused to violate our privacies, to spread misinformation and disinformation, and even be used as a weapon to attack our institutions and destabilize our democratic societies. It is becoming clear that, aside from technology, we also need ethical, legal, and societal innovations to better harness the power and avoid pitfalls of big data. We expect to see more and more research topics like this collection in the future with these diverse topics motivated by societal impact and concerns of big data.

## Author Contributions

All authors listed have made a substantial, direct, and intellectual contribution to the work and approved it for publication.

## Conflict of Interest

NA is employed by the International Business Machines corporation. KW is an employee of Microsoft Research.

The remaining author declares that the research was conducted in the absence of any commercial or financial relationships that could be construed as a potential conflict of interest.

